# PubChem BioAssay: A Decade’s Development toward Open High-Throughput Screening Data Sharing

**DOI:** 10.1177/2472555216685069

**Published:** 2017-01-13

**Authors:** Yanli Wang, Tiejun Cheng, Stephen H. Bryant

**Affiliations:** 1 National Center for Biotechnology Information, National Library of Medicine, National Institutes of Health, Bethesda, MD, USA

**Keywords:** PubChem BioAssay, high-throughput screening, open access, data sharing

## Abstract

High-throughput screening (HTS) is now routinely conducted for drug discovery by both pharmaceutical companies and screening centers at academic institutions and universities. Rapid advance in assay development, robot automation, and computer technology has led to the generation of terabytes of data in screening laboratories. Despite the technology development toward HTS productivity, fewer efforts were devoted to HTS data integration and sharing. As a result, the huge amount of HTS data was rarely made available to the public. To fill this gap, the PubChem BioAssay database (https://www.ncbi.nlm.nih.gov/pcassay/) was set up in 2004 to provide open access to the screening results tested on chemicals and RNAi reagents. With more than 10 years’ development and contributions from the community, PubChem has now become the largest public repository for chemical structures and biological data, which provides an information platform to worldwide researchers supporting drug development, medicinal chemistry study, and chemical biology research. This work presents a review of the HTS data content in the PubChem BioAssay database and the progress of data deposition to stimulate knowledge discovery and data sharing. It also provides a description of the database’s data standard and basic utilities facilitating information access and use for new users.

## PubChem BioAssay：オープンなハイスループットスクリーニングデータ共有に向けた10年にわたる開発

創薬を目的としたハイスループットスクリーニング（HTS）は、今や製薬企業と学術機関および大学のスクリーニング施設の両方で日常的に行われている。アッセイ法の開発、ロボットオートメーション、コンピュータ技術の急速な進歩により、スクリーニング検査室においてテラバイト単位のデータ生成が可能となった。HTS生産性に向けた技術開発が進む一方で、HTSデータの統合および共有に向けた取り組みはほとんど行われなかった結果、膨大な量のHTSデータはほとんど公表されていなかった。この状況を打開するために、PubChem BioAssayデータベース（https://www.ncbi.nlm.nih.gov/pcassay/）が2004年に立ち上げられ、化学物質やRNAi試薬に関するスクリーニング結果に自由にアクセスできるようになった。10年を超える開発および業界による貢献により、PubChemは今や最大規模の化学構造および生物学データの公的なリポジトリとなり、創薬、医薬化学研究、化学生物学研究を支える世界中の研究者達に、情報プラットフォームを提供している。本稿では、知識発見ならびにデータ共有を促すべく、PubChem BioAssayデータベースに含まれるHTSデータの内容とデータ蓄積の進捗状況をレビューする。また、新規ユーザーによる情報へのアクセスおよび使用を容易にする、本データベースのデータ規格および基本ユーティリティについても説明する。

## 퍼브켐 바이오애세이: 오픈 HTS(고속 대량 스크리닝) 데이터 공유를 향한 10년 동안의 개발

HTS(고속 대량 스크리닝)는 이제 제약 회사와 학술 기관 및 대학의 스크리닝 센터에서 약품 발견을 위해 일상적으로 실시됩니다. 분석 개발, 로봇 자동화 및 컴퓨터 기술의 급속한 발전으로 스크리닝 실험실에서 테라바이트의 데이터가 생성되고 있습니다. HTS 생산성 향상을 위한 기술 개발에도 불구하고 HTS 데이터 통합 및 공유에 대한 노력이 줄어 들었습니다. 그 결과 엄청난 양의 HTS 데이터가 거의 대중에게 공개되지 않았습니다. 이러한 격차를 메우고 화학 물질 및 RNAi 시약에 대해 테스트된 스크리닝 결과에 공개적으로 액세스할 수 있도록 2004년 퍼브켐 바이오애세이 데이터베이스(https://www.ncbi.nlm.nih.gov/pcassay/)가 설치되었습니다. 퍼브켐은 커뮤니티에서 10년이 넘는 개발과 기여를 통해 화학 구조 및 생물학 데이터와 관련된 가장 큰 공공 저장소로 진화했으며, 약물 개발, 의약 화학 연구 및 화학 생물학 연구를 지원하는 전세계 연구자에게 정보 플랫폼을 제공합니다. 여기에서는 퍼브켐 바이오애세이 데이터베이스의 HTS 데이터 내용과 정보 검색 및 데이터 공유를 자극하기 위한 데이터 증착 진행 상황에 대해 검토합니다. 또한 신규 사용자가 손쉽게 정보에 액세스하고 사용할 수 있도록 도와주는 데이터베이스의 데이터 표준 및 기본 유틸리티에 대해 설명합니다.

## 公共化合物数据库计划生物鉴定：十年一剑，开启高通量筛查数据共享时代

高通量筛查（HTS）现已经成为各大制药公司和研究机构与高校筛查中心在新药研发中必不可少的技术环节。试验开发、机器人自动化和计算机科技的高速发展，已经开启了筛查实验室兆兆字节数据的新时代。然而，虽然提高HTS效能的创新技术层出不穷，HTS的数据综合与分享技术却几乎无人问津。因此，公众很难接触到海量的HTS数据信息。为填补这一空白，公共化合物数据库计划生物鉴定数据库于https://www.ncbi.nlm.nih.gov/pcassay/2004年正式创立，为公众提供开放渠道以获得有关各类化学制剂和RNAi试剂的筛查结果。经过10年的蓬勃发展，加上来自社区的不懈努力，公共化合物数据库计划现已成为最大的化学结构和生物数据公共储存库，为世界各地从事药物研发、药物化学研究和化学生物研究的研究者们提供了广阔的信息平台。这一成果令我们得以重新审视公共化合物数据库计划生物鉴定数据库中的HTS数据内容和数据存储的进展，从而刺激新的知识发掘和数据共享。它还为新用户提供了数据库的数据标准及其基本应用功能信息的获取和使用说明。

## 公共化合物資料庫計劃生物鑒定：十年一劍，開啟高通量篩查資料共享時代

高通量篩查（HTS）現已經成為各大製藥公司和研究機構與高校篩查中心在新藥研發中必不可少的技術環節。試驗開發、機器人自動化和電腦科技的高速發展，已經開啟了篩查實驗室兆百萬位組資料的新時代。然而，雖然提高HTS效能的創新技術層出不窮，HTS的資料綜合與分享技術卻幾乎無人問津。因此，大眾很難接觸到海量的HTS資料資訊。為填補這一空白，公共化合物資料庫計劃生物鑒定資料庫於(https://www.ncbi.nlm.nih.gov/pcassay/) 2004年正式創立，為大眾提供開放管道以獲得有關各類化學製劑和RNAi試劑的篩查結果。經過10年的蓬勃發展，加上來自社區的不懈努力，公共化合物資料庫計劃現已成為最大的化學結構和生物資料公共儲存庫，為世界各地從事藥物研發、藥物化學研究和化學生物研究的研究者們提供了廣闊的資訊平台。這一成果令我們得以重新審視公共化合物資料庫計劃生物鑒定資料庫中的HTS資料內容和資料儲存的進展，從而刺激新的知識發掘和資料共享。它還為新使用者提供了資料庫的資料標準及其基本套用功能資訊的獲得和使用說明。

## Introduction

High-throughput screening (HTS) is a key technology for drug discovery that allows researchers to test hundreds of thousands of samples.^1^ Screening of diverse libraries of small molecules has proven to be an essential method for identifying chemical starting points for early-stage drug discovery. Functional genomic screening is now often performed using an RNAi reagent library tailored toward a whole genome to identify genes critical to a biological process under study, answering fundamental biological questions and discovering novel therapeutic targets.^2^ Despite being a relatively recent innovation, HTS technology is increasingly empowered by advances in many scientific and technical fields, such as instrumental automation, combinatorial chemical synthesis, and assay technology. It has also been spurred by the breakthrough in biological and genomic research for raising hypothesis and suggesting molecular targets as stimulated by the sequencing of the human genome.^3^

In addition to the technical advances, new and exciting trends are emerging. One such trend is the growing HTS capacity in academic settings,^4,5^ which used to be dominated by industry. As of 2016, more than 100 screening facilities at universities and academic institutions are registered at the Society for Laboratory Automation and Screening (SLAS; http://www.slas.org/resources/information/academic-screening-facilities/). Another trend is the call, support, and implementation for HTS data sharing,^6^ which is widely accepted to be essential for research verification, data reuse, and knowledge discovery. Despite the expanded screening facilities in industry and academia, HTS data sharing was largely lacking. Fortunately, this situation started to change in 2003, given a breakthrough in the open-access movement toward enhancing public access to biomedical research supported by taxpayers. The open-access efforts were led by funding agencies and journal publishers taking steps^7–9^ to mandate the deposition of manuscripts in PubMed Central (PMC; https://publicaccess.nih.gov/policy.htm) and research data in public repositories (https://grants.nih.gov/grants/policy/data_sharing/data_sharing_guidance.htm).

The PubChem project (https://pubchem.ncbi.nlm.nih.gov/)^10^ started in 2004 at the National Center for Biotechnology Information (NCBI) in response to the open-access mandate. The PubChem BioAssay database was set up initially to archive the small-molecule HTS data from the National Institute of Health’s (NIH) Molecular Libraries Program (MLP), which funded a U.S.-wide screening center network between 2004 and 2013 targeting chemical probe development.^11^ It grew tremendously over the past decade in both data capacity and utility,^12^ with assay data contributed by more than 80 organizations and research laboratories. In addition to participation in MLP, PubChem collaborates with other initiatives funded by U.S. government agencies, the European Bioinformatics Institute (EBI), international functional genomics research consortiums, pharmaceutical companies, and journal publishers. For instance, PubChem exchanges small-molecule bioactivity data with ChEMBL,^13^ a chemical biology database hosted by EBI primarily based on literature curation. PubChem also collaborates with several other chemical biology curation databases, such as Guide to PHARMACOLOGY,^14^ BindingDB,^15^ and PDBbind.^16^ Another exemplary collaboration was with the RNAi Global Initiative, which put PubChem in outreach with the research groups conducting RNAi screening in the United States and the European biomedical community. This collaboration led to further development of the BioAssay data model and more than 100 large RNAi datasets in PubChem associated with recent publications. Importantly, many of the RNAi datasets, as well as several small-molecule datasets, were submitted to PubChem as required by journals, representing an excellent demonstration of collective efforts from the funding agencies, screening community, and journal publishers to support open access and HTS data sharing.

Warehousing the big HTS data with a great diversity of assay protocols and making them easily accessible to the public present a big challenge to the development of PubChem. It requires continuous development regarding archival capacity, data model flexibility, and search and analysis utilities to meet the evolving and changing needs from the community.^17–21^ These development efforts were greatly acknowledged, as demonstrated by a recent comprehensive review on the community’s use of the PubChem resources,^12^ which was based on more than a thousand research papers published before 2014 by worldwide researchers telling how the PubChem resource was used in support of their research. The review work showed that the large collection of bioactivity data and molecular target information in PubChem BioAssay had greatly facilitated a number of research areas, such as validating compound bioactivity and target, generating bioactivity profile, virtual screening, polypharmacology research, and drug repositioning. Additionally, it is interesting that a significant number of informatics resources and tools were developed by the community to analyze or annotate the PubChem data, as summarized in the supplementary data of that work.^12^

The PubChem BioAssay resource continues to be explored by the community, as shown by the growing number of citations for the PubChem resource. Interesting and insightful work using PubChem BioAssay is more likely to be found by searching in PubMed or PMC with the keywords “PubChem BioAssay” or otherwise simply with “PubChem” in general. While most of the applications of PubChem BioAssay paid extensive attention to the bioactive compounds and associated targets, the compounds consistently reported with inactive results across the assay collection in PubChem were recently explored for developing good starting point chemicals with unique activity and clean safety profiles,^22^ which illustrated the benefit of archiving inactive data in the public repository. As another example, Helal et al. developed the HTS fingerprints (PubChem HTSFPs) by taking advantage of a large compound library that was tested in hundreds of assays deposited in PubChem BioAssay across a wide panel of targets.^23^ The growth of research development built in part on the PubChem BioAssay resource clearly showed researchers’ recognition of the resource and enthusiasm in data mining and knowledge discovery. Kim et al. recently reported the utilization of the HTS toxicity data in PubChem BioAssay for exploring mechanism profiling of hepatotoxicity.^24^ Additionally, several previous studies using PubChem BioAssay for toxicity prediction were reviewed by Zhu et al.^25,26^ The applications of the PubChem BioAssay data in supporting virtual screening against several biologically critical targets were recently reviewed.^27^

While the reviews showed appreciation for PubChem’s effort, it should be emphasized that the success of PubChem should also be attributed to the community for making this possible by using the resource, providing feedback, and more importantly, sharing research data. This work focuses on the development of PubChem BioAssay regarding HTS data collection. The work reviews the community contribution for data sharing and the progress of data deposition, and summarizes the HTS data content in PubChem BioAssay to illustrate the need for efforts from both PubChem and the community toward building a stronger biomedical information resource. In particular, data generated by the multiple stages of the MLP are analyzed to facilitate access to the chemical probe developments funded by NIH. A brief description about data access and submission is also provided to familiarize new users and depositors with this information resource.

## HTS Data Content

PubChem BioAssay currently contains 1 million bioassay records, 30,000 protein and gene targets, 3 million tested substances, 2 million unique chemical structures, and 200 million bioactivity outcomes. Additional statistics can be found in **Table 1**. More than 95% of the data content in PubChem BioAssay is contributed by the HTS projects of small molecules (**Tables 2** and **3**) or RNAi reagents (**Table 4**) from dozens of worldwide screening facilities at universities, academic institutions, and pharmaceutical companies. Initially, the majority of the HTS data in PubChem was submitted by specialist informatics staff from screening centers. However, “wet laboratory” researchers have recently started to submit their data to PubChem. This recent trend has been in response to meeting the need for open access by journal publishers and funding agencies. A few of the HTS data contributors are referenced below for the purpose of illustrating the efforts and the progress being made by the community for data sharing. The entire list of assay depositors can be found at https://pubchem.ncbi.nlm.nih.gov/sources/.

**Table 1.** PubChem BioAssay Statistics (as of October 10, 2016).

Description	Small-Molecule Assays	RNAi Assays
Assay records (AIDs)	1,218,601	91
Substance samples (SIDs)	3,224,025	352,044
Chemical structures (CIDs)	2,283,536	—
Bioactivity outcomes	230,270,094	1,033,519
Data points	1,499,625,480	14,598,030
Species	3,543	7
Protein targets	10,182	—
Protein targets (human)	4,784	—
Gene targets	—	55,714
Gene targets (human)	—	24,888
Gene targets with phenotype	—	15,866

**Table 2.** Summary of MLP’s HTS Assay Projects.

	Assay Count^a^			Compound Count^b^			
Screening Center	Summary	Primary	Confirmatory	Tested	Active	Chemical Probe	Protein Target Count
Broad Institute	103	136	950	500,665	129,547	27	233
Burnham Center for Chemical Genomics	102	206	651	419,794	143,200	36	450
Columbia University Molecular Screening Center		19	10	197,092	9,067		9
Emory University Molecular Libraries Screening Center	2	22	29	348,780	24,326		20
Johns Hopkins Ion Channel Center	25	103	106	345,281	37,359	4	23
Molecular Libraries Program, Specialized Chemistry Center, University of Kansas	2		22	2,941	312		10
NIH Chemical Genomics Center (NCGC)	179	36	976	443,829	244,064	35	255
New Mexico Molecular Libraries Screening Center (NMMLSC)	30	167	206	375,901	40,549	15	69
Penn Center for Molecular Discovery (PCMD)		26	31	224,377	4,424		16
Southern Research Specialized Biocontainment Screening Center	14	1	272	355,238	16,350	5	11
Southern Research Molecular Libraries Screening Center (SRMLSC)	1	47	40	224,571	31,718	2	11
Scripps Research Institute Molecular Screening Center	150	468	703	397,994	136,876	54	574
University of Pittsburgh Molecular Library Screening Center	1	32	48	223,277	25,711	1	16
Vanderbilt Screening Center for GPCRs, Ion Channels and Transporters	13	15	73	222,812	20,078	6	94
Vanderbilt Specialized Chemistry Center	10	14	125	1,750	683	63	132

a AID count.

b CID count.

**Table 3.** Summary of Small-Molecule HTS Screens (Excluding MLP).

Data Source	Assay Count	Compound Count		Protein Target Count
		Tested^a^	Active	
Abbott Labs	2	7,567	4,912	
ChemBank	106	5,201	1,629	
Chemical genetic matrix	2	13,048	1,568	
Cheminformatics & Chemogenomics Research Group (CCRG), Indiana University School of Informatics	36	2,500	970	
Chen Lab, School of Medicine, Emory University	1	1,947	15	1
Circadian Research, Kay Laboratory, University of California at San Diego (UCSD)	2	1,276	15	
UCLA Molecular Screening Shared Resource	1	1,385	5	
NCI’s Developmental Therapeutics Program (DTP/NCI)	173	176,929	25,036	
GlaxoSmithKline (GSK)	15	14,038	14,038	2
Genomics Institute of the Novartis Research Foundation (GNF)/Scripps Winzeler Lab	1	5,662	274	
Gregory J. Crowther	6	13,451	227	6
ICCB–Longwood/NSRB Screening Facility, Harvard Medical School	28	528,893	10,426	15
Meiler Lab, Vanderbilt University	10	11,385	3,259	4
Milwaukee Institute for Drug Discovery	13	17,808	1,251	1
NCI’s Molecular Targets Development Program (MTDP)	4	99,858	861	4
NINDS Approved Drug Screening Program	34	1,033	190	
NIMH’s Psychoactive Drug Screening Program (PDSP)	2	2,730	603	2
Southern Research Institute	10	361,147	4,871	4
Tox21	105	8,747	4,661	20
UW Madison, Small Molecule Screening Facility	1	69,794	380	
ChEMBL::Novartis Malaria Screening	6	5,614	5,014	
ChEMBL::St. Jude Malaria Screening	16	1,523		

a Only HTS screens testing more than 1,000 samples are included.

**Table 4.** Summary of RNAi HTS Projects.

Data Source	Assay Count	RNAi Reagent Count	Gene Target Count	
			Tested	Show Phenotype
Cancer Research UK Cambridge Research Institute	1	331	331	97
Department of Molecular Cell Biology, Weizmann Institute of Science	1	85	85	20
Drosophila RNAi Screening Center (DRSC)	37	31,356	14,276	3,894
GE Healthcare Dharmacon RNAi Technologies	1	840	840	5
Iain Fraser	14	1,512	252	239
InfectX Consortium	1	115,372	18,612	
INSERM, Institut National de la Sante et de la Recherche Medicale	2	22,950		
Peterson Lab, Genentech	1	158	157	33
siGENOME Human KINOME Library (BTR reporter screen)	1	714	713	49
Genomics Institute of the Novartis Research Foundation (GNF)	1	33,364	17,453	268
Victorian Centre for Functional Genomics, Peter MacCallum Cancer Centre	12	39,160	34,619	3,690
VTT Technical Research Centre of Finland (CSMA)	1	1,380	660	422

As the first depositor of the PubChem BioAssay, the Developmental Therapeutics Program at the National Cancer Institute (DTP/NCI)^28^ shared the anticancer drug screening data on human tumor cell lines, yeast, and mouse models before PubChem made its first public release back in 2004. This contribution greatly helped PubChem in setting up its initial infrastructure and data processing pipeline. The pioneering work of DTP/NCI was followed by more than a dozen screen centers within the MLP,^11^ the NIH’s initiative aimed to develop small-molecule chemical probes for studying the functions of a broad range of proteins and genes. A network of screening facilities at universities and research institutes across the United States, most of which are also listed at SLAS, was funded through two phases of the MLP: the Molecular Libraries Screening Centers Network (MLSCN) and the Molecular Libraries Probe Production Centers Network (MLPCN). As of today, the now ended MLP is still by far the largest HTS data contributor to the PubChem BioAssay database. To facilitate the community’s utilization of the research data generated by the 10-year-long HTS campaign, HTS data generated from the multiple stages of the MLP are summarized in the “MLP’s HTS Data” section.

The Tox21 program (https://www.epa.gov/chemical-research/toxicology-testing-21st-century-tox21), a collaboration between NIH, the Environmental Protection Agency (EPA), and the Food and Drug Administration (FDA), has had more than 100 datasets from about 30 HTS projects deposited in PubChem BioAssay since 2012. The program tests a library of 10,000 compounds covering a broad range of chemicals found in industrial processes, consumer products, food additives, and human and veterinary drugs. It aims to provide evaluation of the chemicals collected regarding their potential and extent for disrupting biological processes in the human body that may lead to adverse health effects.^29–31^ The data generated by the program contains rich information for toxicity evaluation. Novel agonists and antagonists were identified for various biological pathways, such as the retinoic acid receptor (RAR) signaling pathway, NFkB signaling pathway, and endoplasmic reticulum stress response signaling pathway. The Tox21 datasets provide a great opportunity for a comprehensive evaluation of the collected chemicals via the bioactivity and toxicity profile, given a common library that was tested in various pathways similarly to the capacity enabled by the MLP, as discussed later.

The ICCB–Longwood Screening Facility at the Harvard Medical School has led the way in the academic sector supporting HTS data sharing.^32^ It has remained an active PubChem contributor since 2010 and has deposited data from about 30 HTS projects, which cover a wide range of biological targets, as published in recent years. Datasets from several legacy screening programs supported by NIH, such as the NINDS Approved Drug Screening Program, also found PubChem as their home once the program was finalized. The open-access calling was also applauded by pharmaceutical companies. As an example, GlaxoSmithKline (GSK) contributed its antimalaria drug screening data to PubChem early in 2010, and data from another inhibition activity against kinetoplastid parasites, including *Leishmania donovani, Trypanosoma cruzi*, and *Trypanosoma brucei*, in 2015.^33^ It is worth noting that a few datasets associated with recent publications were submitted to PubChem lately by researchers who were making the submissions either to meet the open-access requirement by journals or to support data sharing as a voluntary effort. These datasets cover a study reporting inhibitors against human phosphogluconate dehydrogenase (6PGD) published in *Nature Cell Biology*,^34^ a screen of more than 10,000 compounds against five kinases from *Plasmodium falciparum* published in *PLoS One*, and a research paper published in the *Journal of Biomolecular Screening* reporting an HTS strategy for identifying inhibitors of protein–protein interactions with a library of 60,000 compounds.^35^

The RNAi Global Initiative Consortium (http://www.rnaiglobal.org/) pioneered the effort of sharing RNAi research via the PubChem system by depositing a viability screen of human kinase and cell cycle genes in 2009. The second milestone was set by the Drosophila RNAi Screening Center (DRSC),^36^ a member of the above consortium, which made its first submission in 2011 and since then has remained the largest contributor of RNAi data, with nearly 40 RNAi datasets deposited in PubChem BioAssay. Many of these datasets are primarily associated with publications in prestigious journals such as *Nature, Science, Proceedings of the National Academy of Sciences of the United States of America*, and *Nature Genetics*. The exemplary role of DRSC was quickly followed by others. The Victorian Centre for Functional Genomics at the Peter MacCallum Cancer Centre, also a member of the RNAi Global Initiative Consortium, joined forces and has contributed about a dozen datasets starting in 2014, mostly associated with publications in open-access journals.^37^

Among the development for RNAi data sharing, the third exciting milestone was the deposition of a siRNA circadian assay by researchers at the Genomics Institute of the Novartis Research Foundation (GNF) in 2009.^38^ That submission was made in response to the journal *Cell*’s recommendation of open access to the dataset, which was the first RNAi data deposition in PubChem by researchers in the course of the publication process. This initial step in response to the request by *Cell* has been followed by other international peer-reviewed journals and researchers complying with open-access policies. As a result, about 40 RNAi datasets have been submitted to PubChem, including several genome-wide screens. These datasets are primarily associated with publications (**Suppl. Table S1**) in journals promoting the sharing of valuable scientific datasets, such as *Science Signaling*, a weekly journal by the American Association for the Advancement of Science, and *Scientific Data*, an open-access journal from the Nature Publishing Group. Although the RNAi datasets are small in volume compared with the small-molecule datasets in PubChem, such submissions for functional genomic studies by far surpass the efforts from the chemical biology and medicinal chemistry research community with respect to early engagement, continuity, scale, and journal coverage. The greater response to RNAi data sharing by the community is not surprising given the historical and steady contributions from biologists to the growth of biological and genomic public databases, such as GenBank, GEO, and Expression Atlas. To further encourage and ease RNAi data submission, PubChem coordinates with vendors of siRNA reagents, such as GE Healthcare Dharmacon RNAi Technologies, Qiagen, Life Technologies, Applied Biosystems, and Ambion, for registering their catalogs in PubChem so that assay data can be referenced with the RNAi products. This effort enabled across-assay comparison for an RNAi sample, which is critical for identifying and confirming gene functionality and evaluating off-target effects of the reagent. As an example, the product M-012023-02 from GE Healthcare Dharmacon RNAi Technologies is shown to have been tested in five assays deposited in PubChem with data associated with five publications retrievable using the tool at https://pubchem.ncbi.nlm.nih.gov/assay/bioactivity.html?sid=152150429. PubMed links are provided by the tool via the PubMed icon, which can be followed to further retrieve all the samples and assay data reported in an article. The RNAi vendors could take a further step to link to such PubChem tools from the catalog at the vendor’s website to validate products, aggregate research data, and promote data sharing.

**Figure 1.**
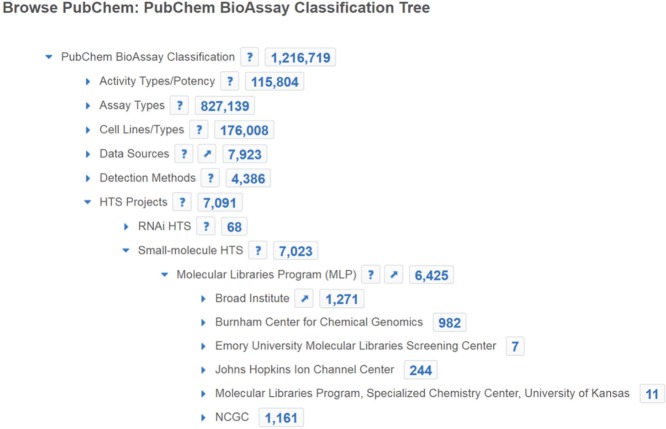
Browse HTS projects using the PubChem BioAssay Classification Tree. A subtree node can be expanded by a click on the triangle icon. The count of BioAssay records associated with each node is shown, and clickable linking to the corresponding list of BioAssay records in Entrez.

Functional genomics plays a crucial role for understanding the dynamic properties of an organism at the cellular level, which is complementary to chemical genomics for drug development by deciphering the responsible biological pathways for a given disease status and suggesting novel drug targets. Whole-genome-based high-throughput RNAi screening is able to rapidly examine each gene in a genome for its potential effect on the phenotype of interest. Having access to both small-molecule and genome-wide screening allows the data integration from both research disciplines and helps to bring together genomic scientists, chemical biologists, and medicinal chemists to synergize discovery efforts. The joint efforts are critical for exploring biological and chemical space effectively to accelerate the identification and validation of drug target, and the understanding of the mode of action for a small molecule as exemplified by the work from Sundaramurthy et al.^39^ While some screening facilities possess both small-molecule and RNAi screening capabilities and others do not, PubChem BioAssay collects, archives, and integrates both types of research results, and enables simultaneous access to functional genomic and chemical genomic HTS data to stimulate the discovery for cross-disciplinary research. Using tools provided at PubChem, one can aggregate RNAi results of a gene that suggest its potential cellular functionality, and meanwhile access the small-molecule bioactivity information to search drugs, chemical probes, agonists, and antagonists that target the same gene (e.g., https://pubchem.ncbi.nlm.nih.gov/assay/bioactivity.html?geneid=659). When starting from a drug molecule, one can combine, compare, and analyze its bioactivity against various protein targets (e.g., https://pubchem.ncbi.nlm.nih.gov/assay/bioactivity.html?cid=9809715) for drug reposition. Both small-molecule and RNAi HTS data can be browsed using the PubChem BioAssay Classification Tree (https://pubchem.ncbi.nlm.nih.gov/assay/assay.cgi?p=classification). For example, as shown in **Figure 1**, one may click to expand the “HTS Projects” node in the tree, and then access RNAi data or link to the more than 6000 datasets from the MLP by clicking on the count. One may further explore the subtree nodes to browse HTS projects from a particular data source, such as the RNAi data deposited by DRSC under “RNAi HTS,” or the small-molecule data from the Tox21 program under “Small-molecule HTS.”

## MLP’s HTS Data

**Figure 2.**
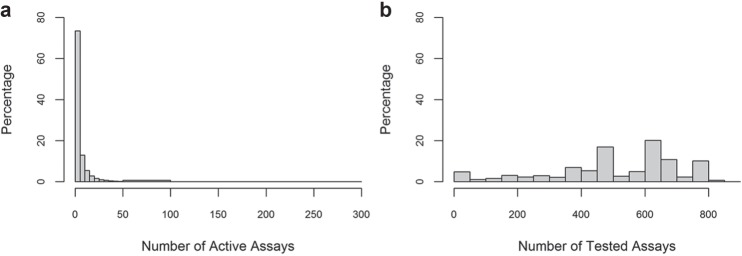
Summary of compounds in the MLSMR library that are associated with biological data. The *x* axis provides a count of BioAssay accessions (AIDs); the *y* axis provides the percentage of the substance samples in MLSMR that are tested across multiple assays at a given count of AIDs. *x* axis for (**a**) counts of all tested assays and for (**b**) counts of only active assays.

Between the fiscal years 2005 and 2014, the MLP carried out more than 600 assay projects and yielded more than 300 chemical probes, along with 6,000 datasets deposited in PubChem BioAssay. Compounds from the NIH’s Molecular Libraries Small Molecule Repository (MLSMR, https://mlsmr.evotec.com/MLSMR_HomePage) were screened across the network of projects. MLSMR grew from 60,000 small molecules in year 2005 to 350,000 by the end of 2009. The library identified and collected compounds from four classes, including specialty sets with known bioactivities, such as drugs, toxins, and metabolites; natural products; targeted libraries with bioactive compounds for protease, kinase, G protein–coupled receptor (GPCR), and so forth; and diversity compounds, with each associated with as many as four close analogs. The structural complexity and diversity of the small molecules in the MLSMR library were analyzed in several studies.^40,41^ At the closure of MLP, 380,000 out of the total of 400,000 compounds in the MLSMR had been tested, in multiple assays, and associated with biological data in PubChem. **Figure 2a** summarizes the association with biological data in PubChem for the compounds in MLSMR, and it can be seen that two-thirds of the small-molecule samples in MLSMR are reported in more than 400 PubChem BioAssay submissions (AIDs). **Figure 2b** shows a similar summary but counts only the active biological test results, providing an estimate for compounds’ “promiscuity” by gathering all active assay data from MLP. The public availability of this large-scale screening campaign using a common library enabled the generation of a biological activity profile and the systematic investigation of biological target space for a large and diversified chemical collection.

**Figure 3.**
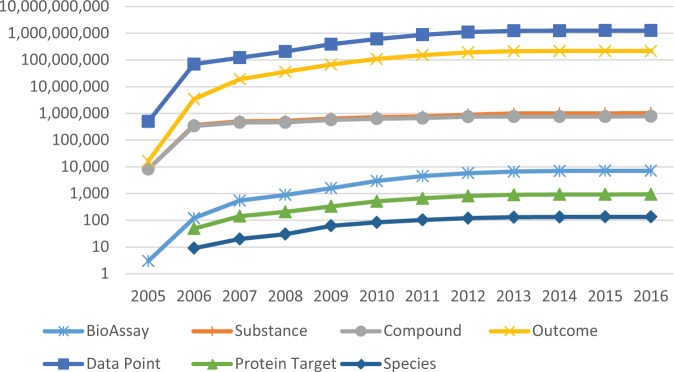
Growth of the MLP’s HTS data, including BioAssay records, tested substances, unique chemical structures, bioactivity outcomes, data points, protein targets, and species.

The growth of the HTS data from MLP is shown in **Figure 3**, including datasets, tested samples, unique chemical structures, bioactivity outcomes, data points, assay targets, and species. More than 4,000 MLP datasets (out of the 6,000 MLP datasets in total) contain biological target specification, while others that do not have molecular target data were either cell based or organism based. Most of the MLP projects started with primary screens using the whole MLSMR library, or a subset of it available at the time of testing. These screens were then followed by multiple dose–response assays for hit confirmation, as well as counterscreens monitoring aspects such as solubility, cytotoxicity, target selectivity, and artifacts. Selectivity screens were often performed against biologically related targets, while toxicity screens were conducted with multiple cell lines. Counterscreens using various assay detection methods were provided as well to rule out false positives. Solubility profiles were generated for the common compound library. As MLP required immediate data deposition, an HTS assay project was often associated with multiple assay submissions as the project advanced and new data were generated. An MLP assay project is represented by a summary AID in the PubChem BioAssay database, and links up all the related datasets deposited over the time reporting the stages of the project, as shown in **Supplementary Figure S1**. Datasets within an assay project may be designated as a group, which is important for interpreting the ultimate outcomes. Such a group of datasets can be accessed from any single assay record within the group through the “Same-Project BioAssays” section on the BioAssay record page (**Suppl. Fig. S1**). Users are highly recommended to utilize and combine the dataset group information, together with other types of related assays for data analysis. A summary of the MLP assay projects and their outcomes is provided in **Table 2**. The summary is provided per each screening center that participated in the network, and it shows that there is a wide range regarding the number of assay projects (represented by summary AIDs in **Table 2**) carried out by the screening centers. The highest productivity was seen for four centers, including the Broad Institute, National Center for Advancing Translational Sciences (NCATS) (formerly NCGC), Burnham Center for Chemical Genomics, and Scripps Research Institute Molecular Screening Center, which in part reflects the funding mechanism that these four screening centers were selected as the “comprehensive centers” at the MLPCN phase for conducting larger-scale HTS projects covering broader research areas.

**Figure 4.**
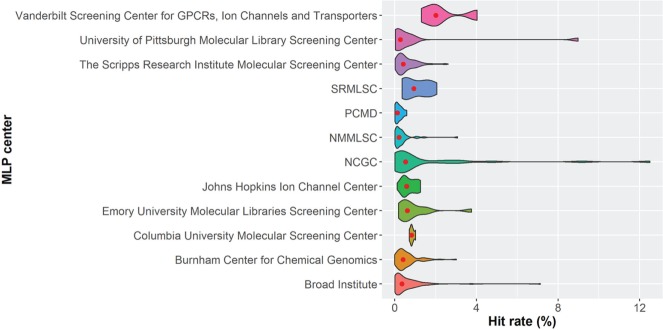
Hit rates for MLP centers. The red dot shows the median of hit rates for each center. Only primary assays that screened more than 100,000 substance samples were included.

The hit rates of the primary screens testing more than 100,000 small-molecule samples for each MLP center are given in **Figure 4**. While the plot shows similar distribution from the four comprehensive screening centers, it is interesting to note the relatively higher hit rates by several specialized centers, such as the Vanderbilt Screening Center for GPCRs, Ion Channels and Transporters, and the Southern Research Molecular Libraries Screening Center (SRMLSC). This is presumably owing to these specialized centers having been more focused on specific research areas and tending to screen targeted compound libraries. For example, the Vanderbilt Screening Center for GPCRs, Ion Channels and Transporters only provided 15 primary screening datasets to PubChem BioAssay, with small-molecule samples no more than 120,000; that is, only a third of MLSMR at most was screened by this center.

**Figure 5.**
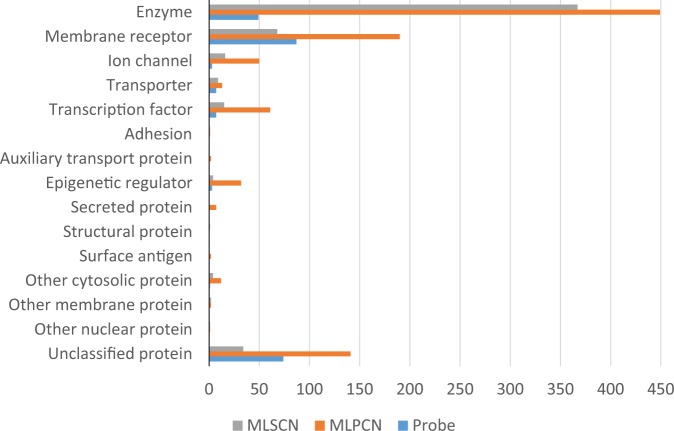
A summary of the MLP assay records (AID count) and chemical probes (probe count) among classes of assay targets. The number of assay records at the two phases of MLP are indicated by MLSCN and MLPCN; the count of chemical probes is indicated by “Probe.”

The MLP has shown great productivity and diversity regarding the coverage of assay target and species, and yields of chemical probes, especially in the MLPCN phase, as shown in **Figure 5** and **Supplementary Figure S2**. A total of 931 unique protein targets, including the primary targets and the biologically related ones in selectivity counterscreens, were tested by the MLP covering a broad range of protein classes, such as enzyme, membrane receptor, and ion channel. The number of the available datasets and chemical probes developed for each protein target class are shown in **Figure 5** for the entire MLP period, as well as partitioned by the MLSCN versus MLPCN phases. The top 20 mostly studied species (134 in total) are given in **Supplementary Figure S2**. More information regarding access to the MLP assay projects, datasets, and chemical probe structures is summarized in **Supplementary Table S2**, offering tips for Entrez search query for identifying specific information regarding the resource generated by MLP. During the course of the chemical probe development, a number of key assay technologies were developed by the MLP. Some of them were recently reviewed, together with a summary of the MLP’s chemical probes,^42^ while others, including the quantitative high-throughput screening (qHTS) technology, which had been employed in almost all the screens carried out by the NIH Chemical Genomics Center (NCGC), were previously described.^43–45^

## Data Presentation, Access, and Submission

PubChem BioAssay implements a one-stop data model with necessary flexibility for accommodating data diversity. An assay record is presented in two parts, including metadata and assay result. The metadata section describes the essential information for an assay, including protocol, molecular target, and cross-reference, and the assay result section reports experimental data linking to the tested samples registered in the PubChem Substance database. The PubChem BioAssay data model allows as many readouts to be reported as needed. Meanwhile, it requires the provision of a “summary result” for each tested substance sample as an indication of bioactivity outcome (e.g., active vs. inactive) and an activity score for ranking hits in a screen. For dose–response test, PubChem requires readout of active concentration, such as IC50 or EC50 (in micromoles) as part of the summary result for small-molecule data. For RNAi data, the gene target of an RNAi reagent is required to be specified. This data standard allows the development of computer tools for data integration and comparison across assay, compound, target, and cell line.^19–21^

Similarly to the MLP datasets, datasets relating to the same assay project generally can be submitted separately to PubChem, making it critical to combine all related datasets for the best interpretation of the underlying data. PubChem BioAssay designates an assay project via the “Summary” assay model, which provides a comprehensive description of the entire project and links to all individual submissions under it. As described above for the MLP data, such related datasets are presented in the “Same-Project BioAssays” section in an assay record page (**Suppl. Fig. S1**) prompting data integration. In general, PubChem supports the designation of related BioAssay records regardless of data source, which allows screens from different laboratories to be linked and compared for research result validation. Via this mechanism, a new assay submission can specify one or multiple AIDs as cross-reference; in turn, PubChem would show a reciprocal relationship from the BioAssay record page of any AID involved. In addition, PubChem BioAssay further derives relationships between the assays based on protein and gene target, common screening library, and same publication.^19–21^ These computational efforts allow search of assays from biologically related targets, such as to find assays containing targets that share protein sequence similarity, or to find assays with targets that have interactions in a biological pathway. They also allow rapid hit evaluation, such as to identify false positives by using related assays from counterscreenings, or to filter out nonspecific hits by looking into common hits across assays. The links between small-molecule datasets and RNAi screening data allow one to combine and accelerate research from multiple scientific disciplines for discovering novel targets for small-molecule drug development, providing chemical tools for further validation of functional genomics study, and deciphering mechanisms of action for small molecules with the integration of RNAi profiling data.

PubChem BioAssay (https://www.ncbi.nlm.nih.gov/pcassay) can be accessed through Entrez, the NCBI information retrieval system. It is cross-linked to other databases in Entrez, such as PubMed, which enables users to access the datasets from the PubMed abstract pages. PubChem BioAssay FTP (ftp://ftp.ncbi.nlm.nih.gov/pubchem/Bioassay/) provides access to all deposited records and derived information. PubChem BioAssay also provides a suite of integrated services (https://pubchem.ncbi.nlm.nih.gov/assay/) enabling users to search, collect, compare, and analyze biological test results. The BioAssay record service provides access to the metadata and entire dataset given the assay accession (AID). As an example, AID 1284, submitted as a dose–response biochemical screen reporting inhibitors of c-Jun N-terminal kinase 3 (JNK3), can be accessed at https://pubchem.ncbi.nlm.nih.gov/bioassay/1284. Additional examples of BioAssay records illustrating various data types with the respective URLs are provided in **Supplementary Table S3**.

Assay data may be submitted via PubChem Upload (https://pubchem.ncbi.nlm.nih.gov/upload/), the PubChem deposition gateway, which provides an extensive set of wizards, in-line help tips, and guided tutorials to assist data submission. Checkpoints for common mistakes are implemented for submission validation to ensure data integrity. PubChem allows depositors to update records and version changes to add, remove, and replace information with all changes archived. PubChem also implements a flexible on-hold mechanism to embargo Substance and BioAssay data to meet special needs from researchers, such as to complete the peer review and publishing process of a journal manuscript, or to wait for the approval of patent application. Additionally, depositors and collaborators have full access to the on-hold data via a secure URL. URLs for important PubChem Upload documents, including login, submission help, FAQs, submission sample files, and guidance for accessing on-hold data are summarized in **Supplementary Table S4**.

## Summary

PubChem BioAssay (https://www.ncbi.nlm.nih.gov/pcassay/) serves as a public repository for archiving biological test results of small molecules and RNAi reagents, which for the first time enabled public access and sharing of large-scale HTS data among the drug discovery and screening community. The complex nature of the HTS data requires a robust information system for tracking data submissions, updates, cross-references, and relationships among datasets. With 12 years’ development and the community’s support, including utilizing the resource, sharing research data, and providing annotations, PubChem has become a widely used public information platform supporting drug development, research for medicinal chemistry, chemical and functional genomics, and bioinformatics and cheminformatics.^12^

The scalable infrastructure built by PubChem as a public archival system is far from being fully utilized by the community for stimulating discovery and supporting data validation, reuse, and interpretation. Researchers’ submission of RNAi data to PubChem is showing the screening community’s support of data sharing. However, progress has been slow and inconsistent, which is similar to a recent finding that data sharing is largely lacking in many research fields for NIH-funded research projects.^46^ On the other hand, there are evolving and positive changes in that funding agencies are tightening up mandatory data sharing policy by explicitly requiring data deposition in public repository when awarding a grant. Meanwhile, awareness from journals and researchers, as well as their support for a data sharing requirement, is increasing, and many open-access journals have been created in recent years calling for data sharing via public repositories. Being now designated as a public repository by a growing list of journals and publishers, PubChem anticipates continuous growth of data deposition in the era of open science. Additionally, a few other areas are under development involving collaborations between PubChem and the community, which include, but are not limited to, the provision of annotations for assay metadata, the validation of assay results, and the development of software tools for annotating assay submission. A stronger public repository for the chemical biology and functional genomics research would also require collaborations among biologists, medicinal chemists, laboratory screeners, and informaticians to further develop ontology and guidelines for describing assay technology, to enhance metadata annotation, and to define practical criteria for hit identification and readout reports. PubChem welcomes and encourages contributions from the SLAS community to use the resource, provide guidance and suggestions, and share research results.

## Supplementary Material

Supplementary material
