# Supplementary material for: PubChem BioAssay: A Decade’s Development toward Open High-Throughput Screening Data Sharing
Source: SLAS Discov. 2017 Jan 13;22(6):655–66. doi: 10.1177/2472555216685069 (PMC5480605; doi:10.1177/2472555216685069)
Supplement: Supplementary material [file MS_revision5.docx]

**PubChem BioAssay: A Decade’s Development Towards Open High-Throughput Screening Data Sharing**

Yanli Wang*, Tiejun Cheng, and Stephen H. Bryant

National Center for Biotechnology Information, National Library of Medicine, National Institutes of Health, Bethesda, MD 20894, USA

*Corresponding author. Email: [ywang@ncbi.nlm.nih.gov](mailto:ywang@ncbi.nlm.nih.gov)

**Abstract**

High-throughput screening (HTS) is now routinely conducted for drug discovery by both pharmaceutical companies and screening centers at academic institutions and universities. Rapid advance in assay development, robot automation, and computer technology have led to the generation of terabytes of data in screening laboratories. Despite the technology development toward HTS productivity, fewer efforts were devoted to HTS data integration and sharing. As a result, the huge amount of HTS data was rarely made available to the public. To fill up this gap, the PubChem BioAssay database (<https://www.ncbi.nlm.nih.gov/pcassay/>) was set up in 2004 to provide open access to the screening results tested on chemicals and RNAi reagents. With over ten years’ development and contributions from the community, PubChem has now become the largest public repository for chemical structures and biological data, which provides an information platform to worldwide researchers supporting drug development, medicinal chemistry study, and chemical biology research. This work presents a review on the HTS data content in the PubChem BioAssay database and the progress of data deposition to stimulate knowledge discovery and data sharing. It also provides a description of the database’s data standard and basic utilities facilitating information access and use for new users.

Keywords: PubChem BioAssay, High throughput screening, Molecular Libraries Program, Data sharing

**Introduction**

High-throughput screening (HTS) is a key technology for drug discovery that allows researchers to test hundreds of thousands of samples^1^. Screening of diverse libraries of small molecules has proven to be an essential method for identifying chemical starting points for early-stage drug discovery. Functional genomic screening is now often performed using RNAi reagent library tailored towards a whole genome to identify genes critical to a biological process under study, answering fundamental biological questions and discovering novel therapeutic targets^2^. Despite being a relatively recent innovation, HTS technology is increasingly empowered by advances in many scientific and technical fields such as instrumental automation, combinatorial chemical synthesis, and assay technology. It has also been spurred by the breakthrough in biological and genomic research for raising hypothesis and suggesting molecular targets as stimulated by the sequencing of human genome^3^.

In addition to the technical advances, new and exciting trends are emerging. One of such trends is the growing HTS capacity in academic settings^4, 5^, which used to be dominated by industry. As of early 2016, over 100 screening facilities at universities and academic institutions are registered at the Society for Laboratory Automation and Screening (SLAS) (<http://www.slas.org/resources/information/academic-screening-facilities/>). Another trend is the call, support, and implementation for HTS data sharing^6^, which is widely accepted to be essential for research verification, data reuse, and knowledge discovery. Despite the expanded screening facilities at industry and academia, HTS data sharing was largely lacking. Fortunately this situation started to change in 2003 given a breakthrough in the open access movement towards enhancing public access to biomedical research supported by taxpayers. The open access efforts were led by funding agencies and journal publishers taking steps^7-9^ to mandate the deposition of manuscript in PubMed Central (<https://publicaccess.nih.gov/policy.htm>) and research data in public repositories (<https://grants.nih.gov/grants/policy/data_sharing/data_sharing_guidance.htm>).

The PubChem project (<https://pubchem.ncbi.nlm.nih.gov/>)^10^ started in 2004 at the National Center for Biotechnology Information (NCBI) in response to the open access mandate. The PubChem BioAssay database was set up initially to archive the small molecule HTS data from the NIH’s Molecular Libraries Program (MLP), which funded a US-wide screening center network between 2004 and 2013 targeting on the chemical probe development^11^. It grew tremendously over the past decade in both data capacity and utility^12^, with assay data contributed by over 80 organizations and research laboratories. In addition to the participation in MLP, PubChem collaborates with other initiatives funded by US government agencies, European Bioinformatics Institute (EBI), international functional genomics research consortiums, pharmaceutical companies, and journal publishers. For instance, PubChem exchanges small molecule bioactivity data with ChEMBL^13^, a chemical biology database hosted by EBI primarily based on literature curation. PubChem also collaborates with several other chemical biology curation databases such as Guide to PHARMACOLOGY^14^, BindingDB^15^, and PDBbind^16^. Another exemplary collaboration was with the RNAi Global Initiative, which put PubChem in outreach with the research groups conducting RNAi screening in the US and the European biomedical community. This collaboration led to further development of the PubChem BioAssay data model and over 100 large RNAi datasets associated with recent publications. Importantly, many of the RNAi datasets as well as several small molecule datasets were submitted to PubChem as required by journals, representing an excellent demonstration of collective efforts from the funding agencies, screening community, and journal publishers to support open access and HTS data sharing.

Warehousing the big HTS data with a great diversity of assay protocols and making them easily accessible to the public present a big challenge to the development of PubChem. It requires continuous development regarding archival capacity, data model flexibility, and search and analysis utilities by catching new technologies to meet the evolving and changing needs from the community^17-21^. These development efforts were greatly acknowledged as demonstrated by a recent comprehensive review on the community’s use of the PubChem resources^12^, which was based on over a thousand research papers published before 2014 by worldwide researchers telling how the PubChem resource was used in support of their research. The review work showed that the large collection of bioactivity data and molecular target information in PubChem BioAssay had greatly facilitated a number of research areas such as validating compound bioactivity and target, generating bioactivity profile, virtual screening, as well as polypharmacology research and drug repositioning. Additionally, it is interesting that a significant number of informatics resources and tools were developed by the community to analyze or annotate the PubChem data as summarized in the supplementary data of that work^12^.

The PubChem BioAssay resource continues to be explored by the community as shown by the growing citations for the PubChem resource. Interesting and insightful work using PubChem BioAssay are more likely to be found by searching in PubMed or PMC with the keyword “PubChem BioAssay” or otherwise simply with “PubChem” in general. While most of the applications of PubChem BioAssay paid extensive attention to the bioactive compounds and associated targets, the compounds consistently reported with inactive results across the assay collection in PubChem were recently explored for developing good starting point chemicals with unique activity and clean safety profiles^22^ , which illustrated the benefit of archiving inactive data in the public repository. As another example, Helal developed the high-throughput screening fingerprints (PubChem HTSFPs) by taking advantage of a large compound library that was tested in hundreds of assays deposited in PubChem BioAssay across a wide panel of targets^23^. The growth of research development built in part upon the PubChem BioAssay resource clearly showed researchers’ recognition of the resource and enthusiasm in data mining and knowledge discovery. Kim et al. recently reported the utilization of the HTS toxicity data in PubChem BioAssay for exploring mechanism profiling of hepatotoxicity^24^. Additionally, several previous studies using PubChem BioAssay for toxicity prediction were reviewed by Zhu et al.^25, 26^ The applications of the PubChem BioAssay data in supporting virtual screening of several biologically critical targets were recently reviewed^27^.

While the reviews showed appreciation for PubChem’s effort, it should be emphasized that the success of PubChem should also be attributed to the community for making this possible by using the resource, providing feedback, and more importantly by sharing research data. This work focuses on the development of PubChem BioAssay regarding HTS data collection. The work reviews the community contribution for data sharing and the progress of data deposition, and summarizes the HTS data content in PubChem BioAssay to illustrate the need for efforts from both PubChem and the community towards building a stronger biomedical information resource. In particular, data generated by the multiple stages of the MLP program is analyzed to facilitate the access to the chemical probe developments funded by NIH. A brief description about data access and submission is also provided to familiarize new users and depositors with this information resource.

**HTS data content**

PubChem BioAssay currently contains 1 000 000 bioassay records, 30 000 protein and gene targets, 3 000 000 tested substances, 2 000 000 unique chemical structures, and 200 000 000 bioactivity outcomes. Additional statistics can be found in Table 1. Over 95% of the data content in PubChem BioAssay are contributed by the HTS projects of small molecules (Table 2 and Table 3) or RNAi reagents (Table 4) from dozens of worldwide screening facilities at universities, academic institutions, as well as pharmaceutical companies. Initially, the majority of the HTS data in PubChem was submitted by specialist informatics staff from screening centers. However, “wet laboratory” researchers have recently started to submit their data to PubChem. This recent trend has been in response to meeting the need for open access by journal publishers and funding agencies. A few of the HTS data contributors are referenced below for the purpose of illustrating the efforts and the progress being made by the community for data sharing. The entire list of assay depositors can be found at <https://pubchem.ncbi.nlm.nih.gov/sources/.>

As the first depositor of PubChem BioAssay, the Developmental Therapeutics Program at the National Cancer Institute (DTP/NCI)^28^ shared the anti-cancer drug screening data on human tumor cell lines, yeast, and mouse models before PubChem made its first public release back in 2004. This contribution greatly helped PubChem in setting up its initial infrastructure and data processing pipeline. The pioneering work of DTP/NCI was followed by over a dozen of screen centers within the MLP^11^, the NIH’s initiative aimed to develop small molecule chemical probes for studying the functions of a broad range of proteins and genes. A network of screening facilities at universities and research institutes across the United States, most of which are also listed at SLAS, was funded through two phases of the MLP program, i.e. the Molecular Libraries Screening Center Network (MLSCN) and the Molecular Libraries Probe Production Center Network (MLPCN). As of today, the now ended MLP program is still by far the largest HTS data contributor for the PubChem BioAssay database. To facilitate community’s utilization of the research data generated by the ten-year long HTS campaign, HTS data generated from the multiple stages of the MLP program are summarized in the “MLP’s HTS Data” section.

The Tox21 program (<https://www.epa.gov/chemical-research/toxicology-testing-21st-century-tox21>), a collaboration between NIH, EPA, and FDA, has had over 100 datasets from about 30 HTS projects deposited in PubChem BioAssay since 2012. The program tests a library of 10 000 compounds covering a broad range of chemicals found in industrial processes, consumer products, food additives, as well as human and veterinary drugs. It aims to provide evaluation of the chemicals collected regarding their potential and extent for disrupting biological process in the human body that may lead to adverse health effects^29-31^. The data generated by the program contains rich information for toxicity evaluation. Novel agonists and antagonists were identified for various biological pathways, such as the retinoic acid receptor (RAR) signaling pathway, NFkB signaling pathway, and endoplasmic reticulum stress response signaling pathway. The Tox21 datasets provide a great opportunity for a comprehensive evaluation of the collected chemicals via the bioactivity and toxicity profile, given a common library that was tested in various pathways similarly to the capacity enabled by the MLP program as discussed later.

The ICCB-Longwood Screening Facility at the Harvard Medical School has led the way in the academic sector supporting HTS data sharing^32^. It remains as an active PubChem contributor since 2010 and has deposited data from about 30 HTS projects, which cover a wide range of biological targets as published in recent years. Datasets from several legacy screening programs supported by NIH, such as the NINDS Approved Drug Screening Program, also found PubChem as their home upon finalizing the program. The open access calling was also applauded by pharmaceutical companies. As an example, GSK, contributed its anti-malaria drug screening data to PubChem early in 2010, and another inhibition activity data against kinetoplastid parasites including *Leishmania donovani*, *Trypanosoma cruzi*, and *Trypanosoma brucei* in 2015^33^. It is worth noting that a few datasets associated with recent publications were submitted to PubChem lately by researchers, who were making the submissions either to meet the open access requirement by journals or to support data sharing as a voluntary effort. These datasets cover a study reporting inhibitors against Human Phosphogluconate dehydrogenase (6PGD) published in Nature Cell Biology^34^, a screen of over 10 000 compounds against five kinases from *Plasmodium falciparum* published in PLOS ONE, and a research paper published in the Journal of Biomolecular Screening reporting an HTS strategy for identifying inhibitors of protein-protein interactions with a library of 60 000 compounds^35^.

The RNAi Global Initiative consortium (<http://www.rnaiglobal.org/>) pioneered the effort of sharing RNAi research via the PubChem system by depositing a viability screen of human kinase and cell cycles genes in 2009. The second milestone was set by the Drosophila RNAi Screening Center (DRSC)^36^, a member of the above consortium, which made its first submission in 2011 and since then has remained as the largest contributor of RNAi data with nearly 40 RNAi datasets deposited in PubChem BioAssay. Many of these datasets are primarily associated with publications in prestigious journals such as Nature, Science, PNAS, and Nature Genetics. The exemplary role of DRSC was quickly followed by others. The Victorian Centre for Functional Genomics at the Peter MacCallum Cancer Centre, also a member of the RNAi Global Initiative Consortium, joined forces and has contributed about a dozen datasets starting in 2014, mostly associated with publications in open access journals^37^.

Among the development for RNAi data sharing, the third exciting milestone was the deposition of a siRNA circadian assay by researchers at the Genomics Institute of the Novartis Research Foundation (GNF) back in 2009^38^. That submission was made in response to the journal Cell’s recommendation of open access to the dataset, which was the first RNAi data deposition in PubChem by researchers in the course of the publication process. This initial step in response to the request by Cell has been followed by other international peer-review journals and researchers complying with open access policies. As a result, about 40 RNAi datasets have been submitted to PubChem, including several genome-wide screens. These datasets are primarily associated with publications (Supplementary Table S1) in journals promoting the sharing of valuable scientific datasets such as Science Signaling, a weekly journal by the American Association for the Advancement of Science, and Scientific Data, an open access journal from the Nature Publishing Group. Though the RNAi datasets are small in volume comparing to the small molecule datasets in PubChem, such submissions for functional genomic studies by far surpass the efforts from the chemical biology and medicinal chemistry research community with respect to early engagement, continuity, scale, and journal coverage. The greater response to RNAi data sharing by the community is not surprising given the historical and steady contributions from biologists to the growth of biological and genomic public databases, such as GenBank, GEO, and Expression Atlas. To further encourage and ease RNAi data submission, PubChem coordinates with vendors of siRNA reagents, such as GE Healthcare Dharmacon RNAi Technologies, Qiagen, Life Technologies, Applied Biosystems, and Ambion for registering their catalogs in PubChem so that assay data can be referenced with the RNAi products. This effort enabled the across-assay comparison for an RNAi sample, which is critical for identifying and confirming gene functionality and evaluating off-target effects of the reagent. As an example, the product M-012023-02 from GE Healthcare Dharmacon RNAi Technologies is shown to have been tested in five assays deposited in PubChem with data associated with five publications retrievable using the tool at <https://pubchem.ncbi.nlm.nih.gov/assay/bioactivity.html?sid=152150429>. PubMed links are provided by the tool via the PubMed icon
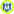
, which can be followed to further retrieve all the samples and assay data reported in an article. The RNAi vendors could take a further step to link to such PubChem tools from the catalog at the vendor’s website to validate products, aggregate research data, and promote data sharing.

Functional genomics plays a crucial role for understanding the dynamic properties of an organism at cellular level, which is complementary to chemical genomics for drug development by deciphering the responsible biological pathways for a given disease status and suggesting novel drug targets. Whole genome based high-throughput RNAi screening is able to rapidly examine each gene in a genome for its potential effect on the phenotype of interest. Having access to both small molecule and genome-wide screening allows the data integration from both research disciplines and helps to bring together genomic scientists, chemical biologist, and medicinal chemists to synergize discovery efforts. The joint efforts are critical for exploring biological and chemical space effectively to accelerate the identification and validation of drug target, and the understanding of the mode of action for a small molecule as exemplified by the work from [Sundaramurthy](https://www.ncbi.nlm.nih.gov/pubmed/?term=Sundaramurthy%20V%5BAuthor%5D&cauthor=true&cauthor_uid=24481274) et al.^39^ While some screening facilities possess both small molecule and RNAi screening capabilities and others do not, PubChem BioAssay collects, archives, and integrates both types of research results, and enables simultaneous access to functional genomic and chemical genomic HTS data to stimulate the discovery for cross-disciplinary research. Using tools provided at PubChem, one can aggregate RNAi results of a gene which suggests its potential cellular functionality and meanwhile access the small molecule bioactivity information to search drugs, chemical probes, agonists, and antagonists that target the same gene (e.g. <https://pubchem.ncbi.nlm.nih.gov/assay/bioactivity.html?geneid=659>). When starting from a drug molecule, one can combine, compare, and analyze its bioactivity against various protein targets (e.g. <https://pubchem.ncbi.nlm.nih.gov/assay/bioactivity.html?cid=9809715>) for drug reposition. Both small molecule and RNAi HTS data can be browsed using the PubChem BioAssay Classification Tree (<https://pubchem.ncbi.nlm.nih.gov/assay/assay.cgi?p=classification>). For example, as shown in Figure 1, one may click to expand the “HTS Projects” node in the tree, then access RNAi data, or link to the over 6 000 datasets from the MLP by clicking on the count. One may further explore the sub-tree nodes to browse HTS projects from a particular data source, such as the RNAi data deposited by Drosophila RNAi Screening Center (DRSC) under “RNAi HTS”, or the small molecule data from the Tox21 program under “Small-molecule HTS“.

**MLP’s HTS Data**

Between the fiscal year 2005 and 2014, the MLP program carried out more than 600 assay projects and yielded over 300 chemical probes along with 6 000 datasets deposited in PubChem BioAssay. Compounds from the NIH’s Molecular Libraries Small Molecule Repository (MLSMR, <https://mlsmr.evotec.com/MLSMR_HomePage>) were screened across the network of projects. MLSMR grew from 60 000 small molecules in year 2005 to 350 000 by the end of 2009. The library identified and collected compounds from four classes including specialty sets with known bioactivities such as drugs, toxins, and metabolites; natural products; targeted libraries with bioactive compounds for protease, kinase, GPCR etc.; and diversity compounds with each associated with as many as four close analogs. The structural complexity and the diversity of the small molecules in the MLSMR library were analyzed in several studies^40, 41^. At the closure of MLP, 380 000 out of the total of 400 000 compounds in the MLSMR have been tested, in multiple assays, and associated with biological data in PubChem. Figure 2a summarizes the association with biological data in PubChem for the compounds in MLSMR, and it can be seen that two thirds of the small molecule samples in MLSMR are reported in over 400 PubChem BioAssay submissions (AID). Figure 2b shows a similar summary but counts only the active biological test results, providing an estimate for compounds’ “promiscuity” by gathering all active assay data from MLP. The public availability of this large-scale screening campaign using a common library enabled the generation of biological activity profile and the systematic investigation of biological target space for a large and diversified chemical collection.

The growth of the HTS data from MLP is shown in Figure 3, including datasets, tested samples, unique chemical structures, bioactivity outcomes, data points, assay targets, and species. Over 4 000 MLP datasets (out of the 6 000 MLP datasets in total) contain biological target specification while others that do not have molecular target data were either cell based or organism based. Most of the MLP projects started with primary screens using the whole MLSMR library, or a subset of it available at the time of testing. These screens were then followed by multiple-dose response assays for hit confirmation, as well as counter screens monitoring aspects such as solubility, cytotoxicity, target selectivity, and artifacts. Selectivity screens were often performed against biologically related targets, while toxicity screens were conducted with multiple cell lines. Counter screens using various assay detection methods were provided as well to rule out false positives. Solubility profiles were generated for the common compound library. As MLP required immediate data deposition, a HTS assay project was often associated with multiple assay submissions as the project advanced and new data was generated. A MLP assay project is represented by a Summary AID in the PubChem BioAssay database, and links up all the related datasets deposited over the time reporting the stages of the project as shown in Supplementary Figure S1. Datasets within an assay project may be designated as a group, which is important for interpreting the ultimate outcomes. Such a group of datasets can be accessed from any single assay record within the group through the “Same-Project BioAssays” section in the BioAssay record page (Supplementary Figure S1). Users are highly recommended to utilize and combine the dataset group information together with other types of related assays for data analysis. Summary of the MLP assay projects and their outcomes are provided in Table 2. The summary is provided per each screening center that participated in the network, and it shows that there is a wide range regarding the number of assay projects (represented by Summary AID in Table 2) carried out by the screening centers. The highest productivity was seen for four centers including Broad Institute, NCATS (formerly NCGC), Burnham Center for Chemical Genomics, and the Scripps Research Institute Molecular Screening Center, which in part reflects the funding mechanism that these four screening centers were selected as the “comprehensive centers” at the MLPCN phase for conducting larger scale HTS projects covering broader research areas.

The hit rates of the primary screens testing over 100 000 small molecule samples for each MLP center are given in Figure 4. While the plot shows similar distribution from the four comprehensive screening centers, it is interesting to note the relative higher hit rates by several centers such as the Vanderbilt Screening Center for GPCRs, Ion Channels and Transporters, and the Southern Research Molecular Libraries Screening Center (SRMLSC). This is presumably owing to these specialized centers having been more focused on specific research areas and tending to screen targeted compound libraries. For example, the Vanderbilt Screening Center for GPCRs, Ion Channels and Transporters only provided 15 primary screening datasets to PubChem BioAssay with small molecule samples no more than 120 000, i.e. only a third of MLSMR at most was screened by this center.

The MLP has shown great productivity and diversity regarding the coverage of assay target and species, and yields of chemical probes, especially in the MLPCN phase as shown in Figure 5 and Supplementary Figure S2. A total of 931 unique protein targets, including the primary targets and the biologically related ones in selectivity counter screens, were tested by the MLP program covering a broad range of protein classes such as enzyme, membrane receptor, and ion channel. The number of the available datasets and chemical probes developed for each protein target class are shown in Figure 5 for the entire MLP program period as well as partitioned by the MLSCN vs. MLPCN phases. The top 20 mostly studied species (134 in total) are given in Supplementary Figure S2. More information regarding the access to the MLP assay projects, datasets, and chemical probe structures are summarized in Supplementary Table S2 offering tips of Entrez search query for identifying specific information regarding the resource generated by MLP. During the course of the chemical probe development, a number of key assay technologies were developed by the MLP program. Some of them were recently reviewed together with a summary of the MLP’s chemical probes^42^, while others including the quantitative high-throughput screening (qHTS) technology, which had been employed in almost all of the screens carried out by the NIH Chemical Genomics Center (NCGC), were previously described^43-45^.

**Data presentation, access, and submission**

PubChem BioAssay implements a one-stop data model with necessary flexibility for accommodating data diversity. An assay record is presented in two parts including metadata and assay result. The metadata section describes the essential information for an assay including protocol, molecular target, and cross-reference, and the assay result section reports experimental data linking to the tested samples registered in the PubChem Substance database. The PubChem BioAssay data model allows as many readouts to be reported as needed. Meanwhile, it requires the provision of a “summary result” for each tested substance sample as an indication of bioactivity outcome (e.g. active vs. inactive) and an activity score for ranking hits in a screen. For dose-response test, PubChem requires readout of active concentration, such as IC50 or EC50 (in micro molar) as part of the summary result for small molecule data. For RNAi data, the gene target of an RNAi reagent is required to be specified. This data standard allows the development of computer tools for data integration and comparison across assay, compound, target, and cell line^19-21^.

Similarly to the MLP datasets, datasets relating to the same assay project generally can be submitted separately to PubChem, making it critical to combine all related datasets for the best interpretation of the underlying data. PubChem BioAssay designates an assay project via the “Summary” assay model, which provides a comprehensive description of the entire project and links to all individual submissions under it. As described above for the MLP data, such related datasets are presented in the “Same-Project BioAssays” section in an assay record page (Supplementary Figure S1) prompting data integration. In general, PubChem supports the designation of related BioAssay records regardless of data source which allow screens from different laboratories to be linked and compared for research result validation. Via this mechanism, a new assay submission can specify one or multiple AID as cross-reference, in turn PubChem would show a reciprocal relationship from the BioAssay record page of any AID involved. In addition, PubChem BioAssay further derives relationship between the assays based on protein and gene target, common screening library, and same publication^19-21^. These computational efforts allow search of assays from biologically related targets, such as to find assays containing targets that share protein sequence similarity, or to find assays with targets that have interactions in a biological pathway. They also allow rapid hit evaluation, such as to identify false positives by using related assays from counter screenings, or to filter out non-specific hits by looking into common hits across assays. The links between small molecule datasets and RNAi screening data allow to combine and accelerate research from multiple scientific disciplines for discovering novel targets for small molecule drug development, providing chemical tools for further validation of functional genomics study, and deciphering mechanism of action for small molecule with the integration of RNAi profiling data.

PubChem BioAssay (<https://www.ncbi.nlm.nih.gov/pcassay/>) can be accessed through Entrez, the NCBI information retrieval system. It is cross-linked to other databases in Entrez, such as PubMed which enables users to access the datasets from the PubMed abstract pages. PubChem BioAssay FTP (<ftp://ftp.ncbi.nlm.nih.gov/pubchem/Bioassay/>) provides access to all deposited records and derived information. PubChem BioAssay also provides a suite of integrated services (<https://pubchem.ncbi.nlm.nih.gov/assay/>) enabling users to search, collect, compare, and analyze biological test results. The BioAssay record service provides access to the metadata and entire dataset given the assay accession (AID). As an example, AID 1284, submitted as a dose response biochemical screen reporting inhibitors of c-Jun N-Terminal Kinase 3 (JNK3), can be accessed at <https://pubchem.ncbi.nlm.nih.gov/bioassay/1284>. Additional examples of BioAssay records illustrating various data types with the respective URLs are provided in Supplementary Table S3.

Assay data may be submitted via PubChem Upload (<https://pubchem.ncbi.nlm.nih.gov/upload/>), the PubChem deposition gateway, which provides an extensive set of wizards, inline help tips, and guided tutorials to assist data submission. Checkpoints for common mistakes are implemented for submission validation to ensure data integrity. PubChem allows depositors to update records and version changes to add, remove, and replace information with all changes archived. PubChem also implements a flexible on-hold mechanism to embargo Substance and BioAssay data to meet special needs from researchers, such as to complete the peer review and publishing process of a journal manuscript, or to wait for the approval of patent application. Additionally, depositors and collaborators have full access to the on-hold data via a secure URL. URLs for important PubChem Upload documents including login, submission help, FAQ, submission sample files, and guidance for accessing on-hold data are summarized in Supplementary Table S4.

**Summary**

PubChem BioAssay (<https://www.ncbi.nlm.nih.gov/pcassay/>) serves as a public repository for archiving biological test results of small molecules and RNAi reagents, which for the first time enabled the public access and sharing of large-scale HTS data among the drug discovery and screening community. The complex nature of the HTS data requires a robust information system for tracking data submission, update, cross-reference, and relationship among datasets. With 12 years’ development and the community’s support including utilizing the resource, sharing research data, and providing annotations, PubChem has become a widely used public information platform supporting drug development, research for medicinal chemistry, chemical and functional genomics, as well as bioinformatics and cheminformatics^12^.

The scalable infrastructure built by PubChem as a public archival system is far from being fully utilized by the community for stimulating discovery and supporting data validation, reuse, and interpretation. Researchers’ submission of RNAi data to PubChem is showing the screening community’s support of data sharing. However, progress has been slow and inconsistent, which is similar to a recent finding that data sharing is largely lacking in many research fields for NIH-funded research projects^46^. On the other hand, there are evolving and positive changes in that funding agencies are tightening up mandatory data sharing policy by explicitly requiring data deposition in public repository when awarding a grant. Meanwhile awareness from journals and researchers as well as their support for data sharing requirement are increasing, and many open access journals have been created in recent years calling for data sharing via public repositories. Being now designated as a public repository by a growing list of journals and publishers, PubChem anticipates continuous growth of data deposition in the era of open science. Additionally, a few other areas are under development involving collaborations between PubChem and the community, which include but not limited to the provision of annotations for assay metadata, validation of assay result, and the development of software tools for annotating assay submission. A stronger public repository for the chemical biology and functional genomics research would also require collaborations among biologists, medicinal chemists, laboratory screeners, and informaticians to further develop ontology and guideline for describing assay technology, enhance metadata annotation, and define practical criteria for hit identification and readout report. PubChem welcomes and encourages contributions from the SLAS community to use the resource, provide guidance and suggestions, and share research results.

**Acknowledgement**

This research was supported by the Intramural Research Program of the National Institutes of Health (NIH), National Library of Medicine (NLM).

**References**

1. Macarron, R.; Banks, M. N.; Bojanic, D.; et al. Impact of high-throughput screening in biomedical research. *Nat. Rev. Drug Discov.* **2011,** *10*, 188-95.

2. Mohr, S. E.; Smith, J. A.; Shamu, C. E.; et al. RNAi screening comes of age: improved techniques and complementary approaches. *Nat .Rev. Mol. Cell Biol.* **2014,** *15*, 591-600.

3. Mayr, L. M.; Bojanic, D. Novel trends in high-throughput screening. *Curr. Opin. Pharmacol.* **2009,** *9*, 580-8.

4. Tralau-Stewart, C. J.; Wyatt, C. A.; Kleyn, D. E.; et al. Drug discovery: new models for industry-academic partnerships. *Drug Discov. Today* **2009,** *14*, 95-101.

5. Frearson, J. A.; Collie, I. T. HTS and hit finding in academia--from chemical genomics to drug discovery. *Drug Discov. Today* **2009,** *14*, 1150-8.

6. Roy, A.; McDonald, P. R.; Sittampalam, S.; et al. Open access high throughput drug discovery in the public domain: a Mount Everest in the making. *Curr. Pharm. Biotechnol.* **2010,** *11*, 764-78.

7. Marta, G. The Access Principle: The Case for Open Access to Research and Scholarship. *Emerg. Infect. Diseases* **2006,** *12*, 1473.

8. Harnad, S.; Brody, T.; Vallières, F.; et al. The Access/Impact Problem and the Green and Gold Roads to Open Access. *Ser. Rev.* **2004,** *30*, 310-314.

9. Butler, D. Scientific publishing: who will pay for open access? *Nature* **2003,** *425*, 554-5.

10. Bolton, E. E.; Wang, Y.; Thiessen, P. A.; et al. Chapter 12 - PubChem: Integrated Platform of Small Molecules and Biological Activities. In *Annu. Rep. Comput. Chem.*; Ralph, A. W.; David, C. S., Eds.; Elsevier: 2008, pp. 217-241.

11. Austin, C. P.; Brady, L. S.; Insel, T. R.; et al. NIH Molecular Libraries Initiative. *Science* **2004,** *306*, 1138-9.

12. Cheng, T.; Pan, Y.; Hao, M.; et al. PubChem applications in drug discovery: a bibliometric analysis. *Drug Discov. Today* **2014,** *19*, 1751-6.

13. Bento, A. P.; Gaulton, A.; Hersey, A.; et al. The ChEMBL bioactivity database: an update. *Nucleic Acids Res.* **2014,** *42*, D1083-90.

14. Southan, C.; Sharman, J. L.; Benson, H. E.; et al. The IUPHAR/BPS Guide to PHARMACOLOGY in 2016: towards curated quantitative interactions between 1300 protein targets and 6000 ligands. *Nucleic Acids Res.* **2016,** *44*, D1054-68.

15. Gilson, M. K.; Liu, T.; Baitaluk, M.; et al. BindingDB in 2015: A public database for medicinal chemistry, computational chemistry and systems pharmacology. *Nucleic Acids Res.* **2016,** *44*, D1045-53.

16. Liu, Z.; Li, Y.; Han, L.; et al. PDB-wide collection of binding data: current status of the PDBbind database. *Bioinformatics* **2015,** *31*, 405-12.

17. Wang, Y.; Xiao, J.; Suzek, T. O.; et al. PubChem: a public information system for analyzing bioactivities of small molecules. *Nucleic Acids Res.* **2009,** *37*, W623-33.

18. Li, Q.; Cheng, T.; Wang, Y.; et al. PubChem as a public resource for drug discovery. *Drug Discov. Today* **2010,** *15*, 1052-7.

19. Wang, Y.; Bolton, E.; Dracheva, S.; et al. An overview of the PubChem BioAssay resource. *Nucleic Acids Res.* **2010,** *38*, D255-66.

20. Wang, Y.; Xiao, J.; Suzek, T. O.; et al. PubChem's BioAssay Database. *Nucleic Acids Res.* **2012,** *40*, D400-12.

21. Wang, Y.; Suzek, T.; Zhang, J.; et al. PubChem BioAssay: 2014 update. *Nucleic Acids Res.* **2014,** *42*, D1075-82.

22. Wassermann, A. M.; Lounkine, E.; Hoepfner, D.; et al. Dark chemical matter as a promising starting point for drug lead discovery. *Nat. Chem. Biol.* **2015,** *11*, 958-66.

23. Helal, K. Y.; Maciejewski, M.; Gregori-Puigjane, E.; et al. Public Domain HTS Fingerprints: Design and Evaluation of Compound Bioactivity Profiles from PubChem's Bioassay Repository. *J. Chem. Inf. Model.* **2016,** *56*, 390-8.

24. Kim, M. T.; Huang, R.; Sedykh, A.; et al. Mechanism Profiling of Hepatotoxicity Caused by Oxidative Stress Using Antioxidant Response Element Reporter Gene Assay Models and Big Data. *Environ. Health Perspect.* **2016,** *124*, 634-41.

25. Zhu, H.; Zhang, J.; Kim, M. T.; et al. Big data in chemical toxicity research: the use of high-throughput screening assays to identify potential toxicants. *Chem. Res. Toxicol.* **2014,** *27*, 1643-51.

26. Shan, C.; Elf, S.; Ji, Q.; et al. Lysine acetylation activates 6-phosphogluconate dehydrogenase to promote tumor growth. *Mol. Cell.* **2014,** *55*, 552-65.

27. Kim, S. Getting the most out of PubChem for virtual screening. *Expert Opin. Drug Discov.* **2016,** *11*, 843-55.

28. Driscoll, J. S. The preclinical new drug research program of the National Cancer Institute. *Cancer Treat. Rep.* **1984,** *68*, 63-76.

29. Judson, R.; Houck, K.; Martin, M.; et al. Analysis of the Effects of Cell Stress and Cytotoxicity on In Vitro Assay Activity Across a Diverse Chemical and Assay Space. *Toxicol. Sci.* **2016,** *153*, 409.

30. Chen, S.; Hsieh, J. H.; Huang, R.; et al. Cell-Based High-Throughput Screening for Aromatase Inhibitors in the Tox21 10K Library. *Toxicol. Sci.* **2015,** *147*, 446-57.

31. Wambaugh, J. F.; Wang, A.; Dionisio, K. L.; et al. High throughput heuristics for prioritizing human exposure to environmental chemicals. *Environ. Sci. Technol.* **2014,** *48*, 12760-7.

32. Tolopko, A. N.; Sullivan, J. P.; Erickson, S. D.; et al. Screensaver: an open source lab information management system (LIMS) for high throughput screening facilities. *BMC Bioinformatics* **2010,** *11*, 260.

33. Pena, I.; Pilar Manzano, M.; Cantizani, J.; et al. New compound sets identified from high throughput phenotypic screening against three kinetoplastid parasites: an open resource. *Sci. Rep.* **2015,** *5*, 8771.

34. Lin, R.; Elf, S.; Shan, C.; et al. 6-Phosphogluconate dehydrogenase links oxidative PPP, lipogenesis and tumour growth by inhibiting LKB1-AMPK signalling. *Nat. Chem. Biol.* **2015,** *17*, 1484-96.

35. Voter, A. F.; Manthei, K. A.; Keck, J. L. A High-Throughput Screening Strategy to Identify Protein-Protein Interaction Inhibitors That Block the Fanconi Anemia DNA Repair Pathway. *J. Biomol. Screen.* **2016**.

36. Flockhart, I. T.; Booker, M.; Hu, Y.; et al. FlyRNAi.org--the database of the Drosophila RNAi screening center: 2012 update. *Nucleic Acids Res.* **2012,** *40*, D715-9.

37. Falkenberg, K. J.; Gould, C. M.; Johnstone, R. W.; et al. Genome-wide functional genomic and transcriptomic analyses for genes regulating sensitivity to vorinostat. *Sci. Data* **2014,** *1*, 140017.

38. Zhang, E. E.; Liu, A. C.; Hirota, T.; et al. A genome-wide RNAi screen for modifiers of the circadian clock in human cells. *Cell* **2009,** *139*, 199-210.

39. Sundaramurthy, V.; Barsacchi, R.; Chernykh, M.; et al. Deducing the mechanism of action of compounds identified in phenotypic screens by integrating their multiparametric profiles with a reference genetic screen. *Nat. Protoc.* **2014,** *9*, 474-90.

40. Dandapani, S.; Marcaurelle, L. A. Grand challenge commentary: Accessing new chemical space for 'undruggable' targets. *Nat. Chem. Biol.* **2010,** *6*, 861-3.

41. Xie, X. Q.; Chen, J. Z. Data mining a small molecule drug screening representative subset from NIH PubChem. *J. Chem. Inf. Model.* **2008,** *48*, 465-75.

42. Schreiber, S. L.; Kotz, J. D.; Li, M.; et al. Advancing Biological Understanding and Therapeutics Discovery with Small-Molecule Probes. *Cell* **2015,** *161*, 1252-65.

43. Inglese, J.; Auld, D. S.; Jadhav, A.; et al. Quantitative high-throughput screening: a titration-based approach that efficiently identifies biological activities in large chemical libraries. *Proc. Natl. Acad. Sci. USA* **2006,** *103*, 11473-8.

44. Walsh, K. B.; Teijaro, J. R.; Rosen, H.; et al. Quelling the storm: utilization of sphingosine-1-phosphate receptor signaling to ameliorate influenza virus-induced cytokine storm. *Immunol. Res.* **2011,** *51*, 15-25.

45. Dillon, M. B.; Bachovchin, D. A.; Brown, S. J.; et al. Novel inhibitors for PRMT1 discovered by high-throughput screening using activity-based fluorescence polarization. *ACS Chem. Biol.* **2012,** *7*, 1198-204.

46. Read, K. B.; Sheehan, J. R.; Huerta, M. F.; et al. Sizing the Problem of Improving Discovery and Access to NIH-Funded Data: A Preliminary Study. *PLOS ONE* **2015,** *10*, e0132735.

Table 1. PubChem BioAssay statistics (as of October 10, 2016)

| Description | Small molecule assays | RNAi assays |
| --- | --- | --- |
| assay records (AID) | 1 218 601 | 91 |
| substance samples (SID) | 3 224 025 | 352 044 |
| chemical structures (CID) | 2 283 536 | - |
| bioactivity outcomes | 230 270 094 | 1 033 519 |
| data points | 1 499 625 480 | 14 598 030 |
| species | 3 543 | 7 |
| protein targets | 10 182 | - |
| protein targets (human) | 4 784 | - |
| gene targets | - | 55 714 |
| gene targets (human) | - | 24 888 |
| gene targets with phenotype | - | 15 866 |

Table 2. Summary of MLP’s HTS assay projects

| Screening Center | Assay Count^a^ | | | Compound Count^b^ | | | Protein Target Count |
| --- | --- | --- | --- | --- | --- | --- | --- |
|  | Summary | Primary | Confirmatory | Tested | Active | Chemical Probe |  |
| Broad Institute | 103 | 136 | 950 | 500 665 | 129 547 | 27 | 233 |
| Burnham Center for Chemical Genomics | 102 | 206 | 651 | 419 794 | 143 200 | 36 | 450 |
| Columbia University Molecular Screening Center |  | 19 | 10 | 197 092 | 9 067 |  | 9 |
| Emory University Molecular Libraries Screening Center | 2 | 22 | 29 | 348 780 | 24 326 |  | 20 |
| Johns Hopkins Ion Channel Center | 25 | 103 | 106 | 345 281 | 37 359 | 4 | 23 |
| Molecular Libraries Program, Specialized Chemistry Center, University of Kansas | 2 |  | 22 | 2 941 | 312 |  | 10 |
| NIH Chemical Genomics Center (NCGC) | 179 | 36 | 976 | 443 829 | 244 064 | 35 | 255 |
| New Mexico Molecular Libraries Screening Center (NMMLSC) | 30 | 167 | 206 | 375 901 | 40 549 | 15 | 69 |
| Penn Center for Molecular Discovery (PCMD) |  | 26 | 31 | 224 377 | 4 424 |  | 16 |
| Southern Research Specialized Biocontainment Screening Center | 14 | 1 | 272 | 355 238 | 16 350 | 5 | 11 |
| Southern Research Molecular Libraries Screening Center (SRMLSC) | 1 | 47 | 40 | 224 571 | 31 718 | 2 | 11 |
| The Scripps Research Institute Molecular Screening Center | 150 | 468 | 703 | 397 994 | 136 876 | 54 | 574 |
| University of Pittsburgh Molecular Library Screening Center | 1 | 32 | 48 | 223 277 | 25 711 | 1 | 16 |
| Vanderbilt Screening Center for GPCRs, Ion Channels and Transporters | 13 | 15 | 73 | 222 812 | 20 078 | 6 | 94 |
| Vanderbilt Specialized Chemistry Center | 10 | 14 | 125 | 1 750 | 683 | 63 | 132 |

^a^ AID count

^b^ CID count

Table 3. Summary of small molecule HTS screens (excluding MLP)

| Data Source | Assay Count | Compound Count | | Protein Target Count |
| --- | --- | --- | --- | --- |
|  |  | Tested^a^ | Active |  |
| Abbott Labs | 2 | 7 567 | 4 912 |  |
| ChemBank | 106 | 5 201 | 1 629 |  |
| chemical genetic matrix | 2 | 13 048 | 1 568 |  |
| Cheminformatics & Chemogenomics Research Group (CCRG), Indiana University School of Informatics | 36 | 2 500 | 970 |  |
| Chen Lab, School of Medicine, Emory University | 1 | 1 947 | 15 | 1 |
| Circadian Research, Kay Laboratory, University of California at San Diego (UCSD) | 2 | 1 276 | 15 |  |
| UCLA Molecular Screening Shared Resource | 1 | 1 385 | 5 |  |
| NCI’s Developmental Therapeutics Program (DTP/NCI) | 173 | 176 929 | 25 036 |  |
| GlaxoSmithKline (GSK) | 15 | 14 038 | 14 038 | 2 |
| GNF / Scripps Winzeler lab | 1 | 5 662 | 274 |  |
| Gregory J. Crowther | 6 | 13 451 | 227 | 6 |
| ICCB-Longwood/NSRB Screening Facility, Harvard Medical School | 28 | 528 893 | 10 426 | 15 |
| Meiler Lab, Vanderbilt University | 10 | 11 385 | 3 259 | 4 |
| Milwaukee Institute for Drug Discovery | 13 | 17 808 | 1 251 | 1 |
| NCI’s Molecular Targets Development Program (MTDP) | 4 | 99 858 | 861 | 4 |
| NINDS Approved Drug Screening Program | 34 | 1 033 | 190 |  |
| NIMH’s Psychoactive Drug Screening Program (PDSP) | 2 | 2 730 | 603 | 2 |
| Southern Research Institute | 10 | 361 147 | 4 871 | 4 |
| Tox21 | 105 | 8 747 | 4 661 | 20 |
| UW Madison, Small Molecule Screening Facility | 1 | 69 794 | 380 |  |
| ChEMBL::Novartis Malaria Screening | 6 | 5 614 | 5 014 |  |
| ChEMBL::St Jude Malaria Screening | 16 | 1 523 |  |  |

^a^ Only HTS screens testing over 1 000 samples are included

Table 4. Summary of RNAi HTS projects

| Data Source | Assay Count | RNAi Reagent Count | Gene Target Count | |
| --- | --- | --- | --- | --- |
|  |  |  | Tested | Show Phenotype |
| Cancer Research UK Cambridge Research Institute | 1 | 331 | 331 | 97 |
| Department of Molecular Cell Biology, Weizmann Institute of Science | 1 | 85 | 85 | 20 |
| Drosophila RNAi Screening Center (DRSC) | 37 | 31 356 | 14 276 | 3 894 |
| GE Healthcare Dharmacon RNAi Technologies | 1 | 840 | 840 | 5 |
| Iain Fraser | 14 | 1 512 | 252 | 239 |
| InfectX Consortium | 1 | 115 372 | 18 612 |  |
| INSERM, Institut National de la Sante et de la Recherche Medicale | 2 | 22 950 |  |  |
| Peterson Lab, Genentech | 1 | 158 | 157 | 33 |
| siGENOME human KINOME library (BTR reporter screen) | 1 | 714 | 713 | 49 |
| The Genomics Institute of the Novartis Research Foundation (GNF) | 1 | 33 364 | 17 453 | 268 |
| Victorian Centre for Functional Genomics, Peter MacCallum Cancer Centre | 12 | 39 160 | 34 619 | 3 690 |
| VTT Technical Research Centre of Finland (CSMA) | 1 | 1 380 | 660 | 422 |

Figure 1. Browse HTS projects using the PubChem BioAssay Classification Tree. A sub-tree node can be expanded by a click on the triangle icon
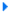
. The count of BioAssay records associated with each node is shown, and clickable linking to the corresponding list of BioAssay records in Entrez.

Figure 2. Summary of compounds in the MLSMR library that are associated with biological data. The X-axis provides a count of BioAssay accessions (AID); the Y-axis provides the percentage of the substance samples in MLSMR that are tested across multiple assays at a given count of AID. X-axis for (a) counts all tested assays; and for (b) counts only active assays.

Figure 3. The growth of the MLP’s HTS data including BioAssay records, tested substances, unique chemical structures, bioactivity outcomes, data points, protein targets and species.

Figure 4. Hit rates for MLP centers. The red dot shows the median of hit rates for each center. Only primary assays that screened over 100 000 substance samples were included.

Figure 5. A summary of the MLP assay records (AID count) and chemical probes (probe count) among classes of assay targets. The number of assay records at the two phases of MLP are indicated by “MLSCN” and “MLPCN”, count of chemical probes is indicated by “Probe”.
